# Medical Education

**Published:** 1881-05

**Authors:** 


MEDICAL EDUCATION.—We invite the special attention and serious consideration of our readers to the paper on this subject in another part of this issue, as further evidence of the importance of a higher standard of qualification for a diploma, and the great necessity of State Medical Examining Boards. The North Carolina Medical Examining Board has furnished pretty conclusive evidence that *vast* and *important differences* should be attached to the value of the diplomas issued by some of the medical schools of this country as indices of the fitness of the holders of them for the duties and responsibilities of practitioners of medicine! It will be seen on referring to the list of the schools, and the number of successful and unsuccessful candidates for license to practice from the North Carolina Examining Board, that the graduates from the schools in this city stand as follows, viz.:

| | | | |
|---|---|---|---|
| University of Maryland, . . | Applied, 8, | . . . . | Licensed, 8. |
| Washington University, . . | " 16, | Rejec'd, 3, | " 13. |
| College Physicians and Surgeons, | " 5, | " 2, | " 3. |
| Non-Graduates. . . . . . | " 4, | " 1, | " 3. |

It will thus be seen that the graduates of our oldest medical school were fully up to the standard required by the Board, whilst those of the last established school *fell below* the applicants who had never graduated at any medical college. This is an unfortunate exhibit, as men of intelligence and attainments in other respects are apt to regard the standing of graduates of a medical school, before seeking a diploma from it, as an endorsement of their professional acquirements.

